# Patient attitudes and preferences about expanded noninvasive prenatal testing

**DOI:** 10.3389/fgene.2023.976051

**Published:** 2023-04-18

**Authors:** Marie-Line Dubois, Patricia D. Winters, Marc-André Rodrigue, Jean Gekas

**Affiliations:** ^1^ Faculty of Medicine, Laval University, Quebec City, QC, Canada; ^2^ Illumina, Inc., San Diego, CA, United States; ^3^ CHU de Quebec Research and Mother and Child Center, Department of Medical Genetics, University Hospital of Quebec, Laval University, Quebec City, QC, Canada

**Keywords:** noninvasive prenatal testing, cell-free DNA, patient preference, surveys and questionnaires, aneuploidy, informed consent, incidental findings

## Abstract

**Introduction:** Noninvasive prenatal testing (NIPT) using cell-free DNA (cfDNA) is typically carried out to screen for common fetal chromosomal anomalies, with the option to screen for a wider range of chromosomal changes (expanded NIPT) becoming increasingly available. However, little is known about pregnant patients’ attitudes and preferences regarding expanded NIPT.

**Methods:** To address this gap, we surveyed general-risk patients having first-tier cfDNA screening at a private prenatal clinic on their expectations for expanded NIPT. Patients were asked questions regarding their current pregnancy and previous pregnancy history, their opinions on fetal DNA screenings during pregnancy and incidental findings, information and opinions on financial resources for NIPT, as well as socio-cultural questions to determine patient demographics.

**Results:** Of the 200 survey participants, the majority were educated, self-reported as white, had a higher than average income, and reported no aneuploidy risk factors. When asked what information they would like to receive from cfDNA screening, the vast majority of participants wanted all information available that could have an immediate impact on fetal health (88%) or an immediate impact on infant health from birth (82%). Many participants also wanted information that could have a future impact on the child’s health or an immediate or future impact on the pregnant woman’s own health. Most participants wanted information about the sex of fetus (86%) and common trisomies (71%), with almost half of participants desiring information about rare autosomal aneuploidies and/or all genetic information that may affect the baby. In addition, participants were found to be comfortable screening for conditions that are well-known, influence care during pregnancy, and are treatable. Finally, while most respondents either had insurance coverage for NIPT or were able to afford NIPT out of pocket, the majority of our participants felt that expanded NIPT should be either free for everyone or for those considered high risk.

**Discussion:** Our findings suggest that with appropriate pre-test counseling, pregnant patients may choose NIPT for an expanding list of conditions.

## Introduction

Noninvasive prenatal testing (NIPT) using cell-free DNA in maternal plasma to screen for fetal aneuploidy was first introduced clinically in 2011. NIPT typically consists of, at a minimum, screening for trisomies 21, 18, and 13, and it is primarily for these trisomies that most practice guidelines recommend screening for all pregnant people (Dondorp et al., 2015; Audibert et al., 2017; American College of Obstetricians and Gynecologists ACOG and Society for Maternal-Fetal Medicine SMFM, 2020; Dungan et al., 2022). A number of professional society guidelines have noted that cfDNA screening is more effective than traditional serum screens in screening for common aneuploidies, with higher sensitivities, specificities, and positive predictive values (PPVs) (Royal College of Obstetricians and Gynaecologists, 2014; Benn et al., 2015; Dondorp et al., 2015; Audibert et al., 2017; American College of Obstetricians and Gynecologists ACOG and Society for Maternal-Fetal Medicine SMFM, 2020; Dungan et al., 2022).

In addition to screening for common trisomies, NIPT for sex chromosome aneuploidies (SCAs) is optional and available in select countries. However, cfDNA screening for common trisomies and SCAs, even with the increased sensitivity of NIPT compared to traditional serum screening options, will miss ∼17% of clinically relevant chromosomal anomalies (Wellesley et al., 2012). In the past few years, the use of NIPT has expanded both in volume and in the number and type of conditions for which screening is available (Ravitsky et al., 2021). The option to screen for additional chromosomal changes, such as rare autosomal aneuploidies (RAAs), select microdeletions, and copy number variants (CNVs) across the genome, collectively referred to as expanded NIPT, is becoming increasingly available through various laboratories.

Several recent publications have shown strong performance for the detection of RAAs and CNVs using expanded NIPT, with high sensitivities, specificities, and low no-call rates observed (Pescia et al., 2017; Pertile et al., 2021; Soster et al., 2021). Some studies have also shown the clinical impact that CNVs and RAAs can have on pregnancy and birth outcomes (Harasim et al., 2022; Mossfield et al., 2022), with the study by van Prooyen Schuurman et al. finding that most of the fetal chromosomal aberrations in their cohort were pathogenic and associated with severe clinical phenotypes (van Prooyen Schuurman et al., 2022). Because NIPT analyzes cfDNA from the placenta and not from the fetus, discordant results due to confined placental mosaicism (CPM) can occur. However, these CPM cases can also be associated with adverse perinatal outcomes (Eggenhuizen et al., 2021; Mossfield et al., 2022; van Prooyen Schuurman et al., 2022). To date, some professional medical societies have remained silent or have recommended against NIPT for RAAs or genome-wide CNVs, mainly citing the lack of large validation studies and the need for further research (Dondorp et al., 2015; Audibert et al., 2017; Kozlowski et al., 2019; American College of Obstetricians and Gynecologists ACOG and Society for Maternal-Fetal Medicine SMFM, 2020). The recent ACMG guidelines note that at this time there is insufficient evidence to either recommend or not recommend noninvasive prenatal screening for the identification of rare autosomal trisomies (Dungan et al., 2022).

Studies exploring patient preferences regarding prenatal screening, and NIPT in particular, have suggested that pregnant patients find NIPT for common aneuploidy screening to be a convenient and safe option that is preferable over conventional serum screening options because of its higher sensitivity and specificity (accuracy) (Farrell et al., 2014a; Lewis et al., 2014; Tiller et al., 2015; Lewis et al., 2016b; Sahlin et al., 2016; Abdo et al., 2018; Bowman-Smart et al., 2019b; Cornell et al., 2022). Fewer studies have explored patient preferences for expanded NIPT. To examine the attitudes and preferences of pregnant people regarding expanded NIPT, we surveyed general-risk patients having first-tier cfDNA screening at a private prenatal clinic in Canada on their expectations for expanded NIPT, including the factors they consider most important when making the decision to undergo expanded cfDNA screening.

## Materials and methods

Pregnant patients presenting to a private prenatal clinic in Quebec City (Prenato Clinics Canada) for consideration of first-tier NIPT for common aneuploidy screening from April 2021 to September 2021 were approached for participation in this study. Inclusion criteria included pregnant patients 18 years of age or older, French-speaking, and ability to provide informed consent for research. We planned to enroll 200 participants. Patients were enrolled on a consecutive basis if they agreed to participate in the study and no advantages were given to participants that agreed to take part in the study. Once enrolled, participants were provided with an informational leaflet (Supplemental Appendix SA [English version]; Supplemental Appendix SB [French version]) describing NIPT, the various types of conditions that could potentially be screened by expanded NIPT, and possible effects of these conditions on the health of the fetus, the pregnancy, the mother, or the child after delivery. If necessary, the patients were free to ask additional questions to the medical team on site. Participants were asked to complete an anonymous electronic survey (Supplemental Appendix SC [English version]; Supplemental Appendix SD [French version]) exploring their attitudes and preferences about expanded NIPT. The survey consisted of a total of 28 questions, covering the following topics: Current pregnancy and pregnancy history; Fetal DNA screenings during pregnancy for the most common trisomies; Additional information that could be accessed through fetal DNA (incidental findings); Financial resources for fetal DNA screenings and incidental findings; and a Socio-cultural section.

Following completion of the survey, participants resumed routine clinical care with a consultation by a clinical nurse to obtain additional information, if needed, and to have blood drawn for NIPT. NIPT offered in this clinic included screening for trisomy 21, trisomy 18, trisomy 13, and sex chromosome aneuploidies (including expected fetal sex). Screening for microdeletions, RAAs, or CNVs was not available at this clinic at the time of the study.

The survey data were analyzed and responses were calculated as percentages. Responses to the Likert-scale questions related to comfort were collapsed into the following three categories: Comfortable (consisting of responses of comfortable and very comfortable), Neutral, and Not Comfortable (consisting of responses of not comfortable and not very comfortable). Responses to Likert-scale questions related to importance were likewise collapsed into the following three categories: Important (consisting of responses of important and very important), Neutral, or Not Important (consisting of responses of not important and not very important). Because of the homogeneity of the data and the small sample size, comparison between response groups was not performed.

## Results

### Participant details

A total of 200 pregnant patients were included in the study cohort. Based on responses to questions 1–6 and 18–28 of the survey (Supplemental Appendix SC), the majority of participants were educated, self-reported as white, and reported no aneuploidy risk factors. Just over half of all participants had at least one child, and around one-quarter of participants had undergone cfDNA screening in a previous pregnancy. In addition, 60% of participants reported an annual family income of greater than $100,000 (see Table 1).

**TABLE 1:** Characteristics of study participants.

Patient characteristics	N (%)
Age, years	
Range	19–40
Median	30
Gestational age, weeks completed	
Range	4–23
Median	12 (SD 2.3)
Previous children	
Yes	102 (51)
No	97 (49)
Previous miscarriage or loss of baby	
Yes	56 (28)
No	143 (72)
cfDNA screening in ≥1 previous pregnancy	
Yes	52 (26)
No or not applicable	147 (74)
Previous pregnancy with a genetic abnormality	
Yes	4 (2)
No	191 (96)
Family history of chromosomal abnormalities	
Yes	15 (8)
No	179 (90)
Method of conception	
Natural	184 (92)
IVF/Assisted reproduction/Other	15 (8)
Country of birth	
Canada	176 (88)
Other	19 (10)
Reported ethnicity	
White	149 (75)
Other	30 (15)
Highest level of education	
High school diploma	12 (6)
College degree	39 (20)
Professional training	20 (10)
Baccalaureate^a^	65 (33)
Master’s degree	37 (19)
Doctorate	13 (7)
Other	8 (4)
Annual family income	
Less than $50,000	12 (6)
$50,001 to $100,000	57 (29)
$100,001 to $300,000	109 (55)
More than $300,000	9 (5)
Religion	
Catholic	108 (54)
Other	25 (13)
No religious affiliation	58 (29)
Considers religion (very) important	
Yes	12 (6)
Neutral	39 (20)
No	138 (69)

^a^ A bachelor’s degree from a university.

Numbers may not total 100% (200) as not all respondents answered every question.

### Desired information from cfDNA screening

**FIGURE 1:**
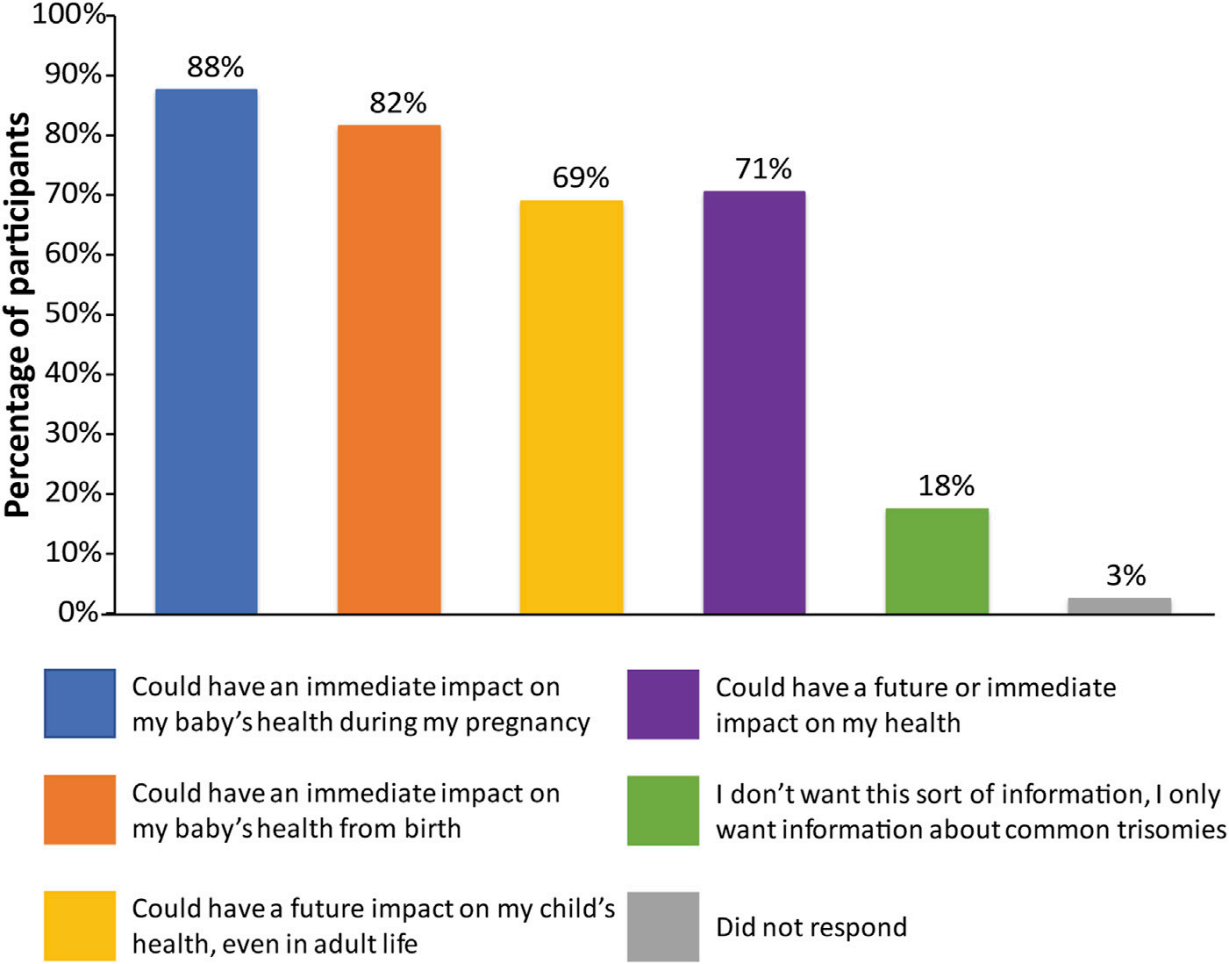
Patient preferences regarding information received from expanded NIPT. Patients were surveyed on the sort of information that they would like to receive with regards to incidental findings, with six options provided. Patients were allowed to select more than one option.

**FIGURE 2:**
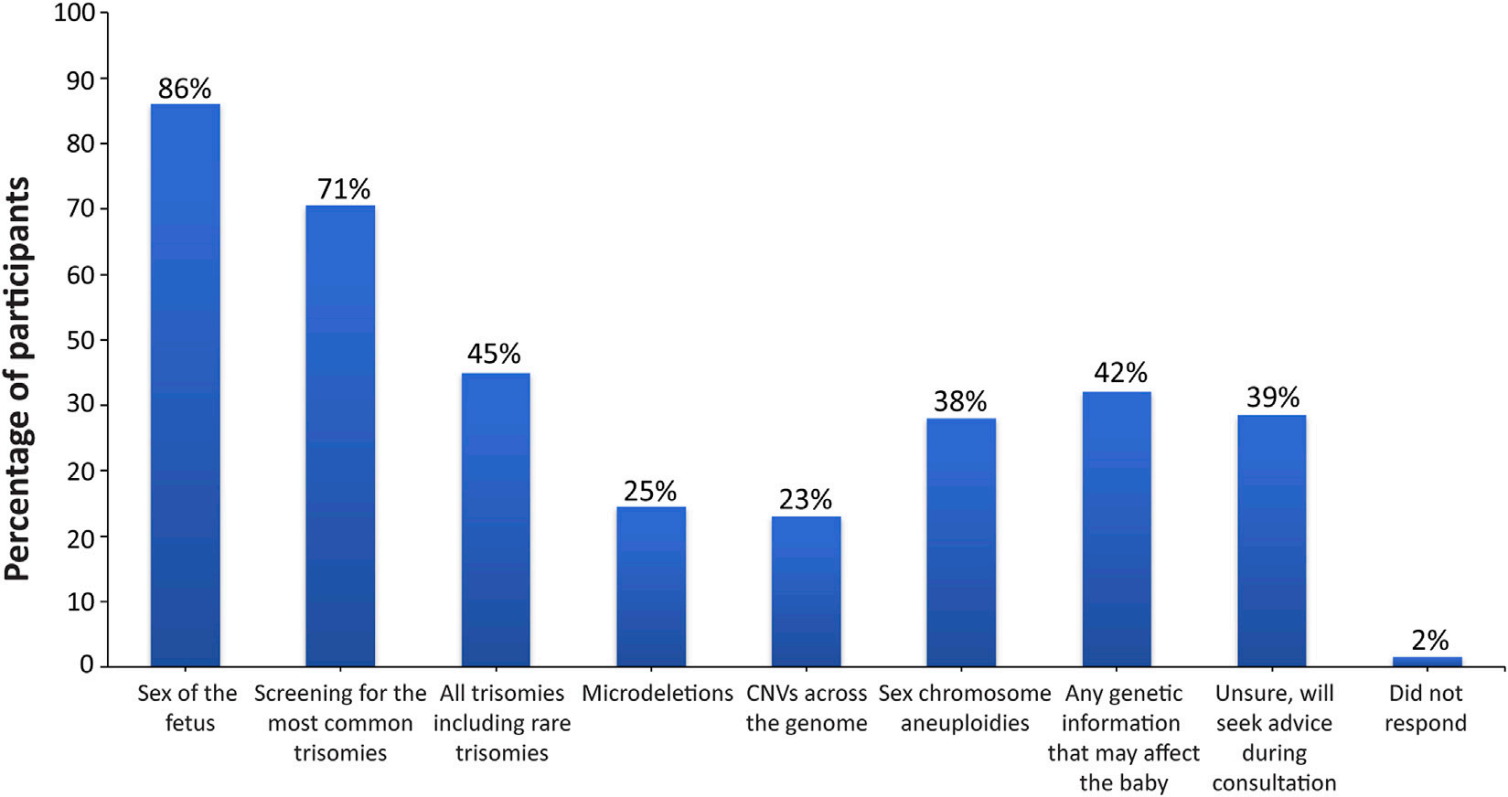
Desired information on the conditions screened for through the fetal DNA test. Patients were surveyed on the information that they would like to receive through the fetal DNA test, with eight options provided. Patients were allowed to select more than one option.

Participants were asked a multi-part question regarding the sort of information that they would be interested in receiving with regard to incidental findings (Supplemental Appendix SC, question 9). The vast majority of participants wanted all information available that could have an immediate impact on fetal health (175; 87.5%) or that had an immediate impact on infant health from birth (163; 81.5%), as shown in Figure 1. Many participants also wanted information that could have a future impact on the child’s health (138; 69%) or an immediate or future impact on the pregnant woman’s own health (141; 70.5%). Only 35 participants (17.5%) did not want information from expanded NIPT and only wanted information about common trisomies. When asked what information they would like to receive through the fetal DNA test (Supplemental Appendix SC, question 7), most participants wanted information about the sex of fetus (172; 86%) and common trisomies (141; 70.5%), as shown in Figure 2. Fewer wanted information about other conditions such as rare trisomies (90; 45%) and CNVs (46; 23%).

### Importance of factors in decision-making

**FIGURE 3:**
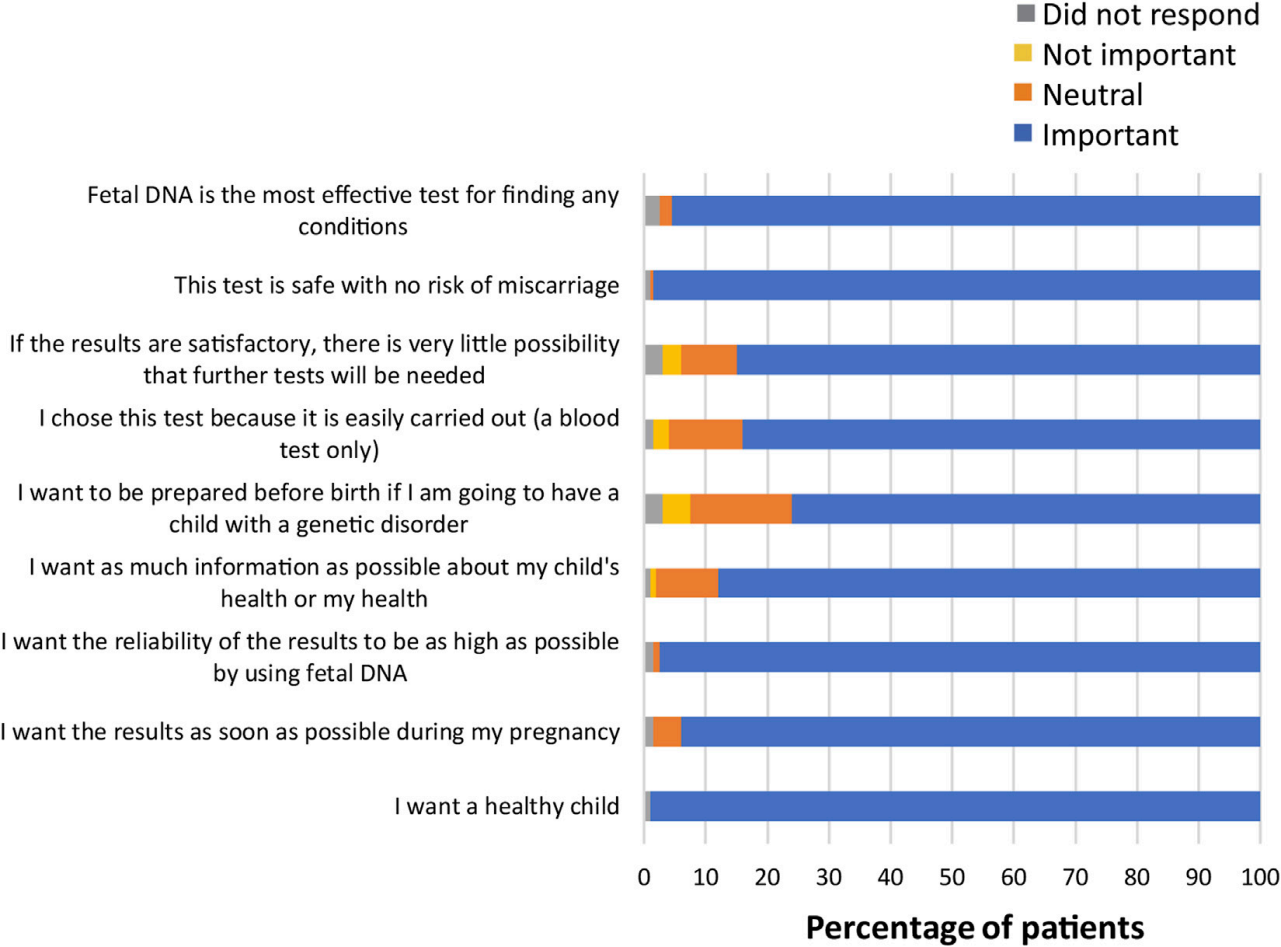
Importance of factors in decision-making for NIPT for common trisomies.

**FIGURE 4:**
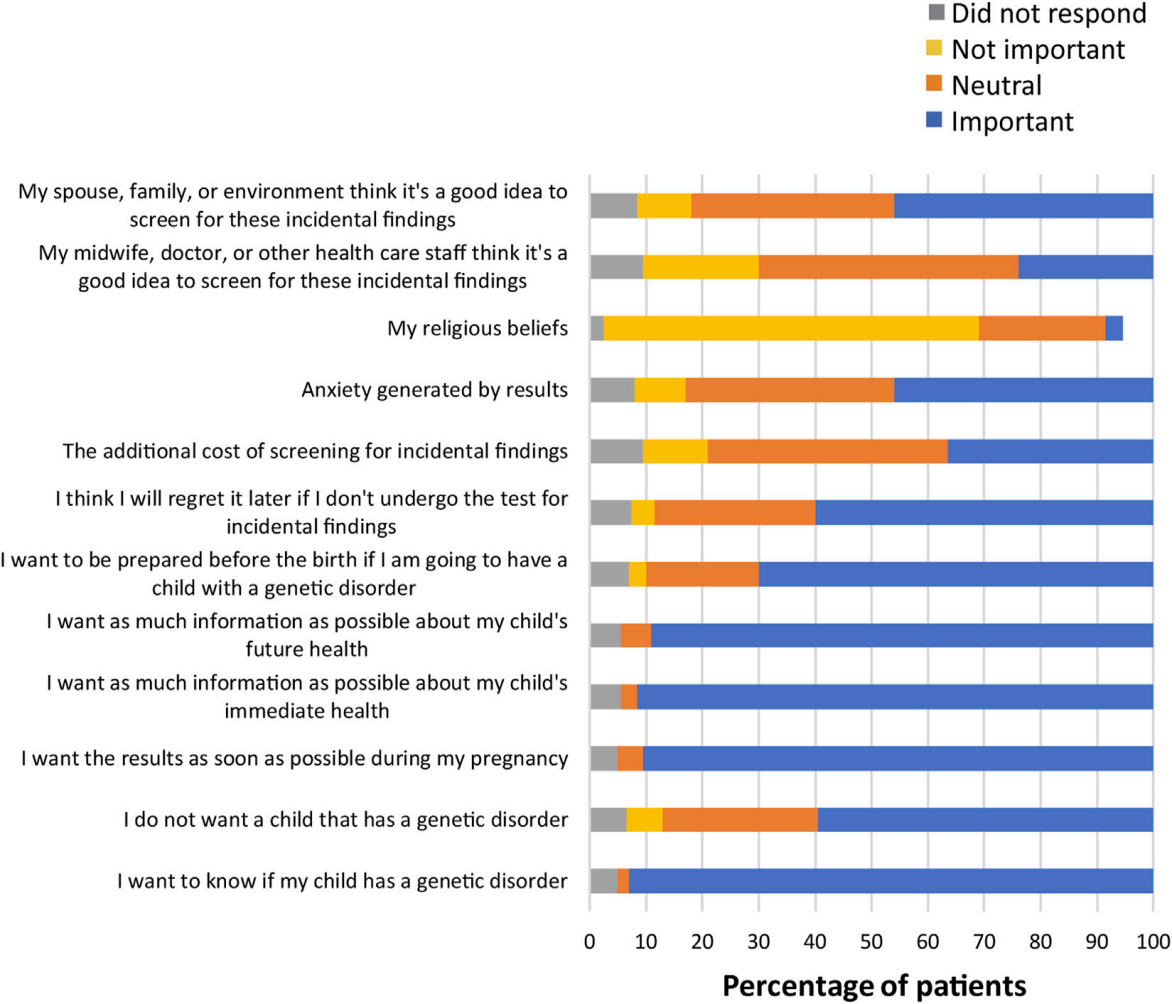
Importance of factors in decision-making for expanded NIPT.

**FIGURE 5:**
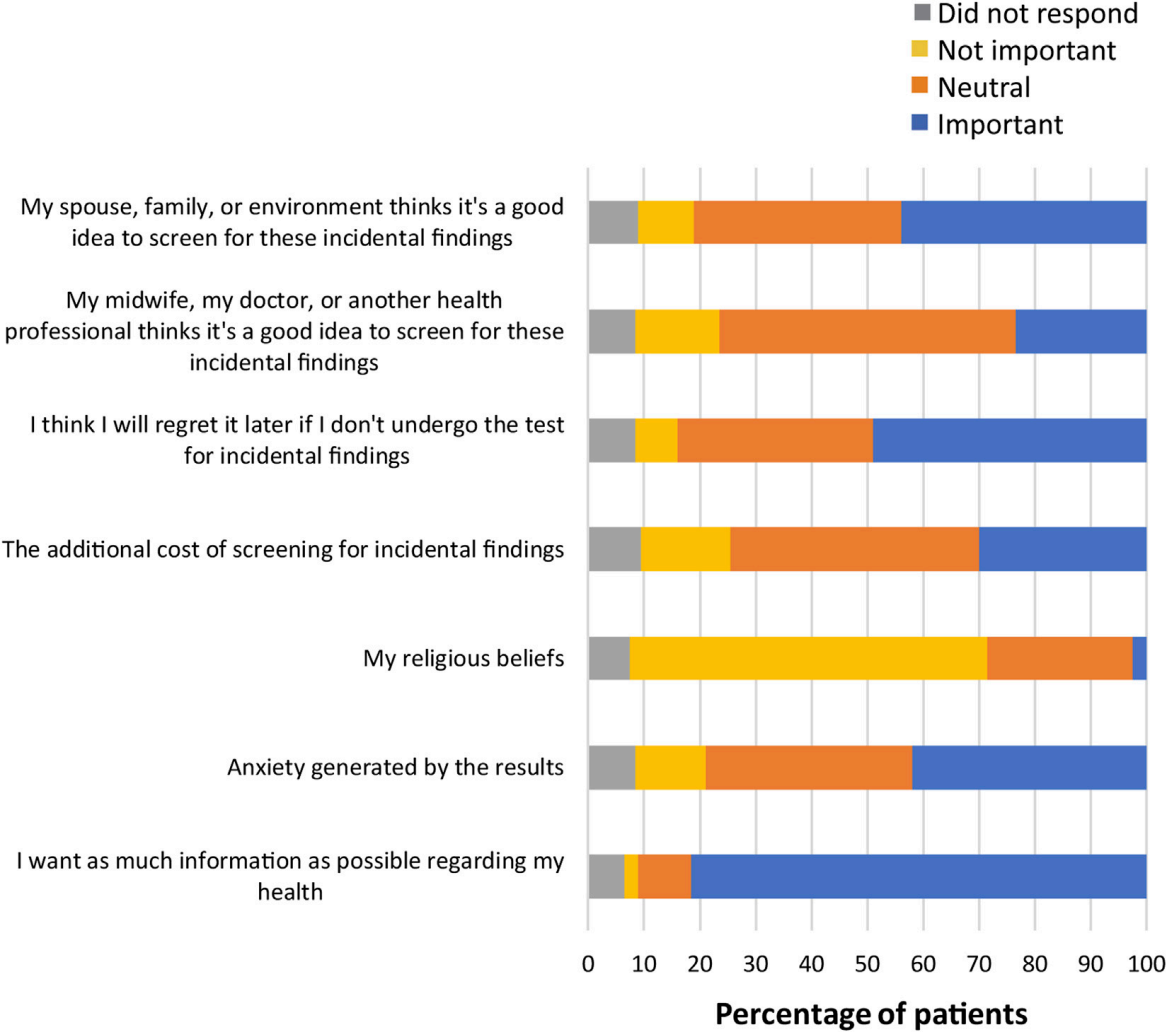
Importance of factors in decision-making for patient’s own health.

Several questions in the survey also asked patients to indicate the importance of various factors in their decision-making process (Supplemental Appendix SC, questions 8, 10, 11). When asked about what factors were most important when making the decision to obtain information about common trisomies, participants responded that wanting a healthy child was important (198; 99%), with 88% of participants (n = 176) stating that wanting as much information as possible about their child’s health or their own health was important (Figure 3). Other factors that were important to almost all participants included having a safe test with no risk of miscarriage (197; 98.5%), wanting the reliability of the results to be as high as possible (195; 97.5%), and that fetal DNA was the most effective test for finding any conditions (191; 95.5%). As can be seen from Figure 4, factors that were most important to participants when making the decision on whether to obtain information from expanded NIPT included wanting to know if their child has a genetic disorder (186; 93%), wanting as much information as possible about their child’s immediate health (183; 91.5%), and wanting as much information as possible about their child’s future health (178; 89%). Wanting the result as soon as possible during the pregnancy was also viewed as important for most participants (181; 90.5%). Participants were then asked about the factors they considered to be important when making decisions about obtaining information regarding their own health (Figure 5). The vast majority responded that wanting as much information as possible about their own health was important (163; 81.5%). Almost half the participants (98; 49%) thought that regretting it later if they did not undergo the test was an important factor in the decision to get information on their own health. Sixty-four percent of participants (n = 128) noted that their religion beliefs were not an important factor in the decision.

### Comfort of participants with information received by expanded NIPT

Participants were also surveyed on their comfort with incidental findings that may involve personal or family risk (Supplemental Appendix SC, question 12). As can be seen from Table 2, participants were found to be comfortable screening for conditions that are well-known (155; 77.5%), influence care during pregnancy (146; 73%), and are treatable (153; 76.5%). Although fewer, participants were still comfortable screening for conditions that are not well known (80; 40%), will not influence care during pregnancy (117; 58.5%), are not treatable (103; 51.5%), and do not appear until adulthood (105; 52.5%).

**TABLE 2:** Participant’s level of comfort with information from expanded NIPT.

	Did not respond, n (%)	Not comfortable, n (%)	Neutral, n (%)	Comfortable, n (%)
Get results that give an assessment of the risks, rather than just a “yes/no” answer	19 (9.5)	31 (15.5)	28 (14.0)	122 (61.0)
Screen for conditions that are well known	21 (10.5)	3 (1.5)	21 (10.5)	155 (77.5)
Screen for conditions that are not well known	25 (12.5)	56 (28.0)	39 (19.5)	80 (40.0)
Screen for conditions that will influence care during pregnancy	23 (11.5)	10 (5.0)	21 (10.5)	146 (73.0)
Screen for conditions that will not influence care during pregnancy	23 (11.5)	13 (6.5)	47 (23.5)	117 (58.5)
Screen for conditions that are treatable	25 (12.5)	2 (1.0)	20 (10.0)	153 (76.5)
Screen for conditions for which there is no treatment	24 (12.0)	37 (18.5)	36 (18.0)	103 (51.5)
If potential disorders do not appear until adulthood	23 (11.5)	24 (12.0)	48 (24.0)	105 (52.5)

### Attitudes of patients regarding coverage for expanded NIPT

**FIGURE 6:**
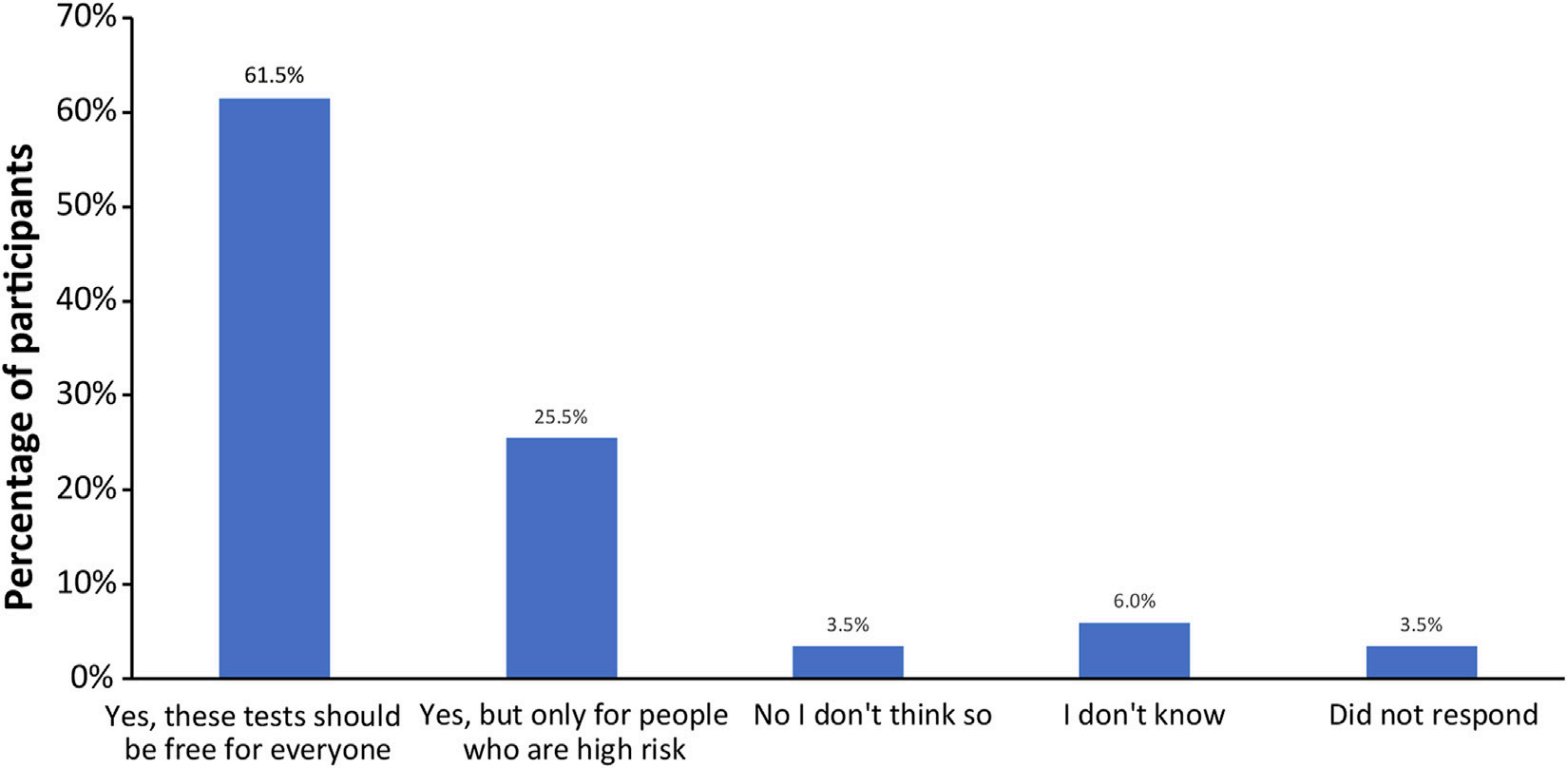
Opinions of participants on whether the public health plan should cover the costs for fetal DNA screening and incidental findings.

**FIGURE 7:**
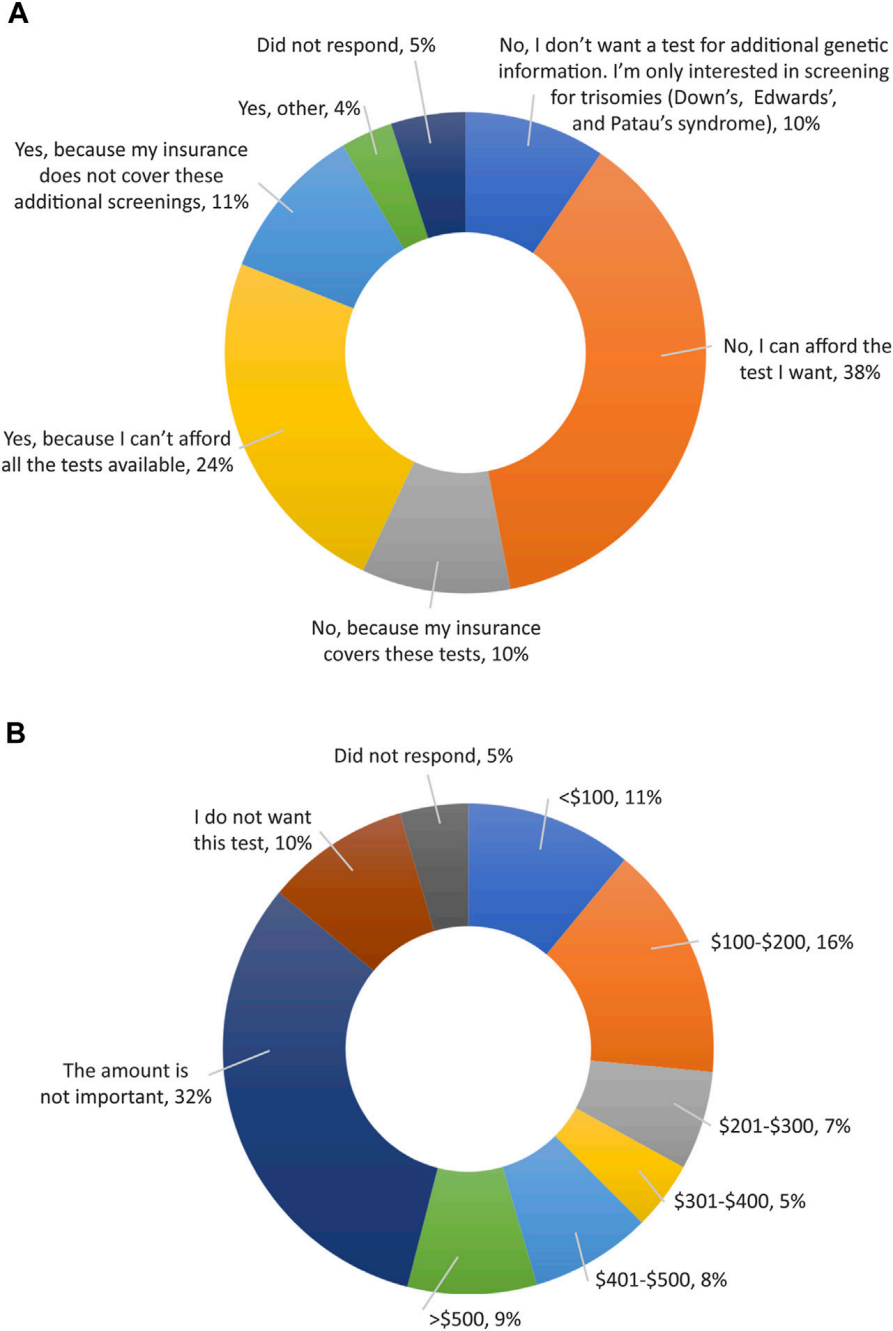
Opinions of participants on reimbursement for expanded cfDNA screening. **(A)** Cost of testing as a factor in a patient’s screening decision. **(B)** Participant’s views on how much they would be willing to pay to access genetic screening for additional information.

Finally, all participants were surveyed on financial resources and reimbursement for fetal DNA screenings and incidental findings (Supplemental Appendix SC, questions 14–17). The majority of participants thought that these tests should be either free for everyone (123; 61.5%) or free for people who are high risk (51; 25.5%), as shown in Figure 6. When asked if cost of testing was a factor in their screening decision (Figure 7A), over half of participants replied “No”, because either their insurance covers these tests (20; 10%); because they can afford the test they want (75; 37.5%); or because they do not want a test for additional genetic information, they are only interested in screening for common trisomies (19; 9.5%). Over a third of participants (71; 35.5%) had insurance that covered at least part of the cost of cfDNA screening. The participants were also asked how much they would be willing to pay for additional findings, with 43% of participants (n = 86) stating that they would be willing to pay at least $100 for it and 32% of participants (n = 64) stating the amount is not important (Figure 7B).

## Discussion

In this study we found that general-risk pregnant people that are undergoing first-tier NIPT, after reading a leaflet with detailed information on the advantages and disadvantages of expanded NIPT screening, are interested in information available through expanded cfDNA screening about both the current and future health of their fetuses and selves. A majority of the survey participants were also comfortable with screening for conditions that will not influence pregnancy care, do not appear until adulthood, or have no treatment. In addition, 42% of participants said they wanted to know any genetic information that could affect the baby.

Most major medical professional societies endorse the option of NIPT to screen for common autosomal aneuploidies (Benn et al., 2015; Dondorp et al., 2015; Audibert et al., 2017; BeSHG, 2017; Salomon et al., 2017; Kozlowski et al., 2019; American College of Obstetricians and Gynecologists ACOG and Society for Maternal-Fetal Medicine SMFM, 2020; BeSHG, 2020; Prieto et al., 2020; Dungan et al., 2022) with some also endorsing cfDNA screening for sex chromosome aneuploidies (Benn et al., 2015; American College of Obstetricians and Gynecologists ACOG and Society for Maternal-Fetal Medicine SMFM, 2020; Dungan et al., 2022). However, as noted above, the use of expanded NIPT has not been endorsed at this time. Our data clearly demonstrate that pregnant patients may be interested in receiving additional findings from expanded NIPT screening. Although the American College of Medical Genetics and Genomics (ACMG) notes that there should be personalized patient-centered counseling (Dungan et al., 2022), and the European Society of Human Genetics/American Society of Human Genetics (ESHG/ASHG) note that pregnant women’s wishes regarding learning information beyond the common trisomies should be taken into account (Dondorp et al., 2015), most guidelines do not acknowledge the preferences of pregnant patients. Our data could thus contribute to a better understanding of patient preferences regarding expanded NIPT and could help to better adapt practice recommendations due to the rapid evolution of genomics, including in the prenatal field, in the near future. In the same way and supporting our results, the Netherlands prenatal screening program, which, since 2017, has offered NIPT with the option of genome-wide expanded options to all pregnant people, emphasizes patient’s opinions and increasing reproductive choices of couples (Bilardo, 2021). A recent publication from this TRIDENT screening program in the Netherlands found that, following a pre-test counseling session with a certified obstetric counselor, 74.2% of patients chose to learn about additional findings other than common trisomies (van Prooyen Schuurman et al., 2022). Another study by the TRIDENT group on patient experiences found that 90.4% of respondents were glad to have been offered the choice between expanded and targeted NIPT, with 76.5% of the respondents choosing to undergo expanded NIPT (van der Meij et al., 2022). The authors concluded that the perspectives of pregnant patients should be included in the dialogue surrounding the expansion of NIPT.

A recent commentary by Bayefsky et al. suggested that criteria used for other health screening programs should be applied to genome-wide NIPT (Bayefsky et al., 2022). These include the condition being an important health problem, that there should be a recognizable latent stage as well as a valid and reliable test and accepted treatment for the condition, and the screening should be cost effective. The authors question whether NIPT should be applied for certain rare conditions if they are not a common cause of disease and disability in the general population. From our perspective, we believe that the prevalence of RAAs and CNVs is high enough to warrant screening for these additional fetal anomalies. In addition, recent publications have shown that the presence of RAAs and CNVs can impact both pregnancy and birth outcomes, and that measures taken during the pregnancy such as increased monitoring can be beneficial and should be considered (Mossfield et al., 2022; van Prooyen Schuurman et al., 2022). We therefore believe it is important that patients are offered the choice of having expanded noninvasive prenatal screening, provided they receive appropriate pretest counselling.

A number of previous studies have looked at patient preferences regarding conventional noninvasive prenatal screening. Here, when participants were asked about the conditions that they desired information on, the vast majority of participants (86%) wanted information on fetal sex, and 71% wanted information on common trisomies. This differs from a 2019 study (Bowman-Smart et al., 2019b) based on survey responses of 235 pregnant patients in Australia, which found that less than a third wanted to undergo NIPT for fetal sex, whilst 86% of respondents noted detection of chromosomal abnormalities as a reason for undergoing NIPT. A study by Farrell et al. (Farrell et al., 2014b) looking at the perspectives of 53 people that were either pregnant or had recently delivered found that accuracy, early timing, ease of testing, and fetal sex determination were the main advantages of NIPT. The recent study by van der Meij et al. noted the main reasons that participants chose expanded NIPT were ‘wanting as much information as possible about the health of the child’ and wanting ‘to be prepared for everything’ (van der Meij et al., 2022). Other studies that have looked at patient expectations and preferences from expanded NIPT often focus on conditions that are not currently available as part of routine cfDNA screening. Most of these studies have shown strong support for including predicted fetal sex (Bowman-Smart et al., 2019a; Haidar et al., 2021). A sizeable portion of pregnant patients are also interested in other expanded NIPT options, including sex chromosomal aneuploidy (Agatisa et al., 2015; Bowman-Smart et al., 2019a), microdeletions (Agatisa et al., 2015; Calonico et al., 2016; Farrell et al., 2016), childhood onset conditions (whether treatable or not) (Farrell et al., 2014a; Sullivan et al., 2019), and conditions of adult-onset (whether preventable or not) (Farrell et al., 2014a; Bowman-Smart et al., 2019a). However, most pregnant patients do not appear to be supportive of using NIPT for non-medical traits, other than fetal sex (Kooij et al., 2009; Bowman-Smart et al., 2019a; Haidar et al., 2021). A 2015 study (van Schendel et al., 2015) of 381 women who completed an online questionnaire on a Dutch website found that the vast majority of participants agreed with screening for a broad range of conditions including severe life-threatening disorders with no available treatment and disorders for which the child can already be treated during pregnancy such as heart disease. The study by van der Meij et al. also noted that most of the respondents were favorable toward a broader future screening offer such as screening for severe untreatable life-threatening disorders, disorders characterized by a mental disability, disorders that can be treated during pregnancy, and severe physical disabilities (van der Meij et al., 2022).

One disadvantage of a screening test is the low PPV that may be associated with it, which can lead to increased patient anxiety. PPV is the proportion of positive results that are truly positive and incorporates test sensitivity and specificity as well as the population prevalence of the condition. This can also lead to unnecessary invasive diagnostic procedures, which are associated with additional risks and costs. Studies looking at expanded NIPT have noted different PPVs for these additional findings; reasons for differing PPVs between studies may include differences in sequencing depth, the background risk profile of the population (e.g., proportion of advanced maternal age), differences in inclusion criteria for study participants, and whether maternal CNVs are included as true positives in the analysis. A recent study looking at test performance of genome-wide cfDNA screening in a real clinical population (Soster et al., 2021) found high sensitivity and specificity, with a PPV of 22.4% for rare autosomal trisomies and 72.6% for genome-wide CNVs. The study also found that 25% of the positive results would have been missed with traditional cfDNA screening. A recent study from the TRIDENT-2 group noted PPVs of 7.7% for rare autosomal trisomies and 44.1% for structural chromosomal aberrations (van Prooyen Schuurman et al., 2022). The 2017 Society of Obstetricians and Gynaecologists of Canada (SOGC) guidelines on prenatal screening for fetal aneuploidy in singleton pregnancies (Chitayat et al., 2017) note that any prenatal screen offered to Canadian women must have a detection rate of 75% with no more than 3%–5% false-positive rate, dependent on trimester of screening. With this scope, a long-time serum screening testing has been used as the preferred prenatal screening program while its PPV was as low as 3%–5%. Nevertheless, for 46% of our patients, the anxiety generated by the results is considered important in decision making when considering the use of an expanded screening test.

Regarding financial resources for NIPT, over 62% of our study participants thought that NIPT should be free for everyone and another 26% thought that is should be free for people with a high-risk pregnancy. Another study (Birko et al., 2019) carried out in Canada a few years ago that also looked at patient attitudes toward NIPT coverage noted similar results, with 67% of pregnant women responding that all patients should have access to NIPT free of charge and 30% saying that only patients with a high-risk pregnancy should be eligible. Studies of healthcare providers in Europe (Benachi et al., 2020), as well as the Lebanon and Quebec (Haidar et al., 2020), found that one of the primary barriers to uptake of NIPT was the cost and lack of reimbursement. Cost and insurance coverage were also noted as disadvantages of NIPT in a study by Farrell et al.(Farrell et al., 2014b) of patients in a clinic in the United States. According to the SOGC, as of January 2021, the cfDNA test is not publicly funded for all pregnant patients in Canada as first-line prenatal screening and is a self-paid or insurance-covered option for most pregnant people if they are not detected at risk by a first-step serum screen. In some Canadian provinces, there is funding for people who meet certain high-risk criteria (Wou et al., 2021). Our results show that a large proportion of patients in a financially well-off population are willing to cover the additional costs related to obtaining additional findings from expanded cfDNA screening.

With increasing use of NIPT, concerns over the potential for ‘routinization’ of prenatal screening have arisen (Lewis et al., 2016a; Cernat et al., 2019). While this concern lacks empirical confirmation in practice (Kater-Kuipers et al., 2018), the importance for patients to have sufficient understanding of prenatal screening options to allow for an informed choice consistent with one’s values is well-recognized. Global medical societies emphasize the need for appropriate pre-test counseling (Benn et al., 2015; Dondorp et al., 2015; Rose et al., 2020; Dungan et al., 2022). Patients, however, have expressed feeling dissatisfied with both the quality and type of information available about NIPT, citing lack of provider knowledge and time constraints (Cernat et al., 2019). As the menu options for NIPT grow and include the potential for conditions with less well-defined phenotypes, reduced penetrance, variable expressivity, and/or later onset, pretest counseling will become more complex and difficult. In particular, understanding the positive predictive value of results for conditions of varying prevalence will be crucial. Pregnant patients, with their partners, can have different preferences for whether NIPT should consist of a fixed set of conditions or whether they should be able to decide which specific condition(s) to screen for in their pregnancy (van Schendel et al., 2014). ACMG recommends discussing the types of conditions that can and cannot be screened using NIPT as part of pretest counselling (Dungan et al., 2022). In addition, it is important to note that genomic abnormalities detected during NIPT may be of parental origin and may indicate a maternal health condition such as maternal malignancy (Turriff et al., 2022; van Prooyen Schuurman et al., 2022). It is important that patients are counselled on the possibility of these additional findings; in our study, most women indicated that they were interested in receiving information that had either an immediate or future impact on their own health.

A limitation of our study is the lack of diversity amongst participants. The majority of patients that carried out the survey self-identified as white, were intermediate to highly educated, were born in Canada, and had an annual family income of greater than $100,000. All participants also had to be French-speaking as the survey was carried out in French. This population largely reflects the population of Quebec City in eastern Canada, which uses the services of a private clinic for pregnancy monitoring, while free prenatal screening programs exist in the province of Quebec. It is therefore possible that the findings from our study may not translate into other more heterogeneous populations such as patients from a lower socio-economic background and different cultural groups. Future studies that are carried out in different regions and that include participants from different educational backgrounds and different socioeconomic backgrounds would be useful for comparison with the results observed in our study population. Another limitation is that the survey includes the opinions of a relatively small number of pregnant patients and did not include opinions of their partners or healthcare providers. However, in our study cohort, only a quarter of participants thought that the opinion of their healthcare provider was an important factor in their decision to screen for incidental findings.

Another limitation of the study was that patients were not fully informed on all of the conditions that can be screened for with genome-wide NIPT, the sensitivity and specificity of NIPT for these conditions, or the limitations of genome-wide NIPT, which may have impacted their responses. The informational leaflet did not contain any information regarding the limitations of expanded NIPT or provide details on the expected PPVs for the different fetal anomalies that are screened for by both traditional and expanded NIPT. It also did not give details on potential reasons for discordant results such as CPM, and the need for additional confirmatory follow-up testing that should be carried out following a positive NIPT result before any decisions regarding the pregnancy are taken. The participants took the survey before their routine consultation with a clinical nurse, and so their responses were based on the information provided in the leaflet. However, the participants were informed that they were also free to ask questions to the medical staff following reading of the brochure if they needed further information. It is possible that this was not sufficient information for them to make a truly informed choice when it came to the different questions in the survey. For example, some patients may not have been aware that presence of a rare autosomal trisomy or CNV could have an immediate impact on fetal health. This may help explain some of the contradictory responses from the participants, such as the fact that while most participants wanted all information available that could have an immediate impact on fetal health, 45% and 23% of women did not want information on rare trisomies and CNVs, respectively. In addition, the multiple-choice answering format of the survey could have contributed to these seemingly contradictory results. A knowledge evaluation of the patients could be carried out in the future to assess the patient knowledge of expanded NIPT following completion of the survey. In addition, the survey did not include any questions relating to patient anxiety associated with a false-positive result or the false reassurance associated with a false-negative result.

In summary, our findings suggest that with appropriate pre-test counseling, pregnant patients may choose NIPT for an expanding list of conditions. However, patients should be made aware of both the benefits and limitations of expanded NIPT and the potential for discordant results. It is very important that appropriate post-test counselling is provided in cases of a high-risk screening result before any decisions on the pregnancy are undertaken. Our results indicate that women can provide their perspective on their preferences on expanded NIPT screening. This study adds to the growing body of research looking at the attitudes, experiences, and opinions of pregnant patients on cfDNA screening, which can be used to inform future policies around the implementation, availability, and scope of this screening technology.

## Data Availability

All of the data supporting the findings in this study are available upon reasonable request from the corresponding author.
