# Supplementary material for: Patient attitudes and preferences about expanded noninvasive prenatal testing
Source: Front Genet. 2023 Apr 18;14:976051. doi: 10.3389/fgene.2023.976051 (PMC10161390; doi:10.3389/fgene.2023.976051)
Supplement: Supplementary file 1 [file DataSheet2.pdf]

Bienvenu chez Prenato et merci d'avoir accepté de remplir ce court questionnaire qui nous permettra de mieux connaître nos patientes afin d'offrir un service toujours à l'avant-garde de la médecine moderne et surtout à votre écoute.

Test de dépistage et test diagnostic génétiques

Un test de dépistage prénatal, à visée génétique, a pour but d'identifier les bébés ayant un risque important d'être porteur d'une maladie génétique. Les résultats obtenus lors d'un test de dépistage ne donne pas de réponse définitive, mais ils permettent d'identifier les patientes à risque pour lesquelles des tests complémentaires, cette fois-ci diagnostics, permettront de déterminer avec certitude si l'enfant est atteint d'une maladie génétique ou chromosomique.

Les tests diagnostics qui confirment, hors de tout doute raisonnable, la présence ou l'absence d'anomalies génétiques se font généralement par l'analyse des cellules de bébé après amniocentèse par exemple. C'est le résultat de ces tests diagnostics qui déterminera avec certitude la santé génétique du bébé. Cependant, ces tests diagnostics sont associés à un certain risque de complication pour le fœtus et c'est pourquoi cette procédure est réservé pour les femmes présentant un risque lors des tests de dépistages.

Maladies génétiques

Il faut mentionner que la grande majorité des enfants qui naissent au Québec sont en bonne santé. Cependant, une petite proportion nait avec des problèmes de santé génétique qui peuvent parfois être détecté pendant la grossesse par différents tests.

Habituellement, chacune des cellules humaines possèdent 23 paires de chromosomes pour un total de 46 dont la dernière paire est composée des chromosomes sexuels (XX pour une femme et XY pour un homme). Les maladies génétiques peuvent être le résultat d'une anomalie de nombre de ces chromosomes (un chromosome en plus ou en moins réalisant une trisomie ou une monosomie) ou parfois une anomalie de la morphologie de l'un de ces chromosomes, par la perte d'un segment de chromosome (délétion) ou encore l'addition d'une partie seulement d'un chromosome (duplication réalisant un excès ou trisomie partielle).

Les anomalies les plus communes (fréquence) et les plus connues sont les trisomies 13, 18 et 21 ainsi que les anomalies de nombre des chromosomes sexuels :

Parfois, une trisomie peut être dépistée pour un chromosome ne faisant pas partie des trisomies communes, ces trisomies dites rares, sont aussi intéressantes à rechercher, car elles peuvent avoir un pronostic sombre, si finalement, elles touchent le bébé ou définir un risque pour la grossesse (ex ; retard de croissance) si elles sont présente dans le placenta du bébé. Le pronostic associé à ces trisomies est moins bien connu que celui des trisomies communes.

Enfin, certains syndromes sont aussi accessibles au dépistage prénatal. Ceux-ci, correspondent à déséquilibre pour un segment de chromosome réalisant un déséquilibre génomique avec un risque conséquent d'enfant à naître avec une déficience génétique.

Tests de dépistage par ADN fœtal

Les tests de dépistage par ADN fœtal utilise l'ADN du fœtus qui se retrouve dans le sang de la mère pour quantifier le nombre de chromosomes. Au moment du dépistage par ADN fœtal, il est laissé, aux parents ou aux professionnels de santé requérant le test, une totale liberté sur le type de dépistage qui sera réalisé sur le prélèvement de sang de la maman. Ainsi, le test de dépistage par ADN fœtal pourrait :

- Soit dépister uniquement les trisomies communes (chromosomes 13, 18 et 21);
- Soit aussi inclure l'identification du sexe du bébé et le dépistage des anomalies des chromosomes sexuels;
- Soit rechercher aussi les trisomies plus rares, concernant les autres paires chromosomiques qui peuvent avoir des conséquences sur l'évolution de la grossesse ou la santé du bébé;
- Soit finalement, analyser la morphologie des chromosomes pour certains syndrome microdélétionnels accessibles au dépistage et responsables de déficiences génétiques de l'enfant à la naissance, si elles sont retrouvées.

Trouvailles fortuites

Généralement, l'ensemble des anomalies génétiques offertes au dépistage et déjà citées ci-dessus, vont se traduire par des troubles du bébé visibles pendant la grossesse ou à la naissance, néanmoins, dans certains cas, une anomalie génétique dépistée pendant la grossesse pourrait seulement se révéler au cours de l'enfance, voir même susciter des troubles seulement à un âge adulte avancé, avec la possibilité d'un traitement médical ou non. Ces situations, avec l'avancé rapide de la génétique moderne devraient devenir plus fréquentes dans l'avenir, ce qui explique certaines questions de notre questionnaire car, votre avis est précieux et il est bien important d'écouter les parents pour adapter au mieux les outils de la médecine de demain.

Welcome to Prenato and thank you for agreeing to complete this short questionnaire which we need to get to know our patients better in order to offer a service that is always at the forefront of modern medicine and, above all, listening to you.

Genetic screening test and diagnostic test

A genetic prenatal screening test aims to identify babies at significant risk of carrying a genetic disease. The results obtained during a screening test do not give a definitive answer, but they make it possible to identify patients at risk for whom additional tests, this time diagnoses, will make it possible to determine with certainty whether the child is affected. genetic or chromosomal disease.

Diagnostic tests that confirm, beyond a reasonable doubt, the presence or absence of genetic abnormalities are usually done by analyzing baby cells after amniocentesis, for example. It is the result of these diagnostic tests that will determine with certainty the genetic health of the baby. However, these diagnostic tests are associated with a certain risk of complications for the fetus and that is why this procedure is reserved for women at risk during screening tests.

Genetic diseases

It should be mentioned that most children born in Quebec are in good health. However, a small proportion are born with genetic health problems that can sometimes be detected during pregnancy by different tests.

Usually, each human cell has 23 pairs of chromosomes for a total of 46 of which the last pair is made up of sex chromosomes (XX for a woman and XY for a man). Genetic diseases can be the result of an abnormality in the number of these chromosomes (one more or less chromosome making a trisomy or a monosomy) or sometimes an abnormality in the morphology of one of these chromosomes, by the loss of 'a chromosome segment (deletion) or even the addition of only part of a chromosome (duplication producing an excess or partial trisomy).

The most common (frequency) and well-known anomalies are trisomies 13, 18 and 21 as well as anomalies in the number of sex chromosomes:

Sometimes a trisomy can be screened for a chromosome that is not part of the common trisomies, these so-called rare trisomies are also interesting to look for, because they can have a poor prognosis, if ultimately, they affect the baby or define a risk for the pregnancy (ex: growth retardation) if they are present in the baby's placenta. The prognosis associated with these trisomies is less well known than that of common trisomies.

Finally, some syndromes are also accessible for prenatal screening. These correspond to an imbalance for a segment of the chromosome producing a genomic imbalance with a consequent risk of an unborn child with a genetic deficiency.

Fetal DNA testing

Fetal DNA testing uses DNA from the fetus in the mother's blood to quantify the number of chromosomes. At the time of fetal DNA screening, parents or health professionals requesting the test are completely free to choose the type of screening that will be carried out on the mother's blood sample. Thus, the fetal DNA test could:

- Or only screen for common trisomy (chromosomes 13, 18 and 21).

- Or include the identification of the sex of the baby and screening for abnormalities of the sex chromosomes.

- Or also look for the rarer trisomy, concerning the other chromosome pairs which can have consequences on the progress of the pregnancy or the health of the baby.

- Or finally, analyze the morphology of chromosomes for certain microdeletal syndrome accessible to screening and responsible for genetic deficiencies in the child at birth, if they are found.

Fortuitous finds
Generally, all the genetic abnormalities offered for screening and already mentioned above, will result in visible disorders of the baby during pregnancy or at birth, however, in some cases, a genetic abnormality detected during pregnancy could only reveal themselves during childhood, or even cause disorders only in late adulthood, with the possibility of medical treatment or not. These situations, with the rapid advance of modern genetics, should become more frequent in the future, which explains some of the questions in our questionnaire because, your opinion is precious, and it is very important to listen to the parents in order to best adapt them. tools of tomorrow's medicine.
