# Supplementary material for: Patient attitudes and preferences about expanded noninvasive prenatal testing
Source: Front Genet. 2023 Apr 18;14:976051. doi: 10.3389/fgene.2023.976051 (PMC10161390; doi:10.3389/fgene.2023.976051)
Supplement: Supplementary file 2 [file DataSheet4.pdf]

Prenato Patient Survey

1. À quelle semaine de grossesse êtes-vous :

2. Avez-vous déjà des enfants

- Oui
- Non
- Si oui, combien :

3. Lors de vos précédentes grossesses avez-vous fait un dépistage par ADN fœtal

- Oui, lors de toutes mes grossesses
- Oui, mais pas pour toutes mes grossesses
- Non
- Ne s’applique pas

4. Avez-vous déjà fait une fausse couche ou perdu un bébé?

- Non
- Oui

5. Cette grossesse a été conçu de manière :

- Naturelle
- Par fertilisation in vitro (IVF)
- Avec une technologie de reproduction assisté autres que (IVF)
- Autre:

6. Indiquez si ces situations s’appliques à vous :

	Non	Oui
Les résultats de l’échographie du premier ou deuxième trimestre indiquaient qu’il était préférable de poursuivre les investigations génétiques	○	○
Je vais avoir 35 ans ou plus lors de l’accouchement	○	○
J’ai eu un test de dépistage sanguin qui indiquait qu’l était préférable de poursuivre les investigations génétiques	○	○
J’ai eu une grossesse antérieure ou il y avait présence d’anomalie génétique	○	○
Mon partenaire ou moi-même avons une anomalie chromosomique ce qui augmente nos chances d’avoir un enfant avec des changements chromosomiques	○	○
Mon partenaire ou moi-même avons une historique familiale d’anomalies chromosomique	○	○
J’ai une condition qui me prédispose à avoir un enfant avec des anomalies chromosomiques (si oui précisez)	○	○

Précisions :

Dépistages par ADN fœtal durant la grossesse pour les trisomies communes 13, 18, 21

7. Quelles informations désirez-vous recevoir via le test d’ADN fœtal? (Vous pouvez cocher plusieurs possibilités)

- ☐ Le sexe de l’enfant
- ☐ Le dépistage des trisomies les plus communes (trisomies 13,18 et 21) seulement
- ☐ Toutes les trisomies incluant celles pour les chromosomes qui sont plus rarement impliquées (trisomies rares)
- ☐ Toutes les microdélétions
- ☐ Tous les déséquilibres génomiques (appelés aussi CNV)
- ☐ Les anomalies des chromosomes sexuels car parfois elles influencent la santé du bébé
- ☐ Je veux obtenir toute l’information génétique pouvant avoir une influence sur le bébé
- ☐ Je ne suis pas encore certaine et vais demander conseil pendant ma consultation avec l’infirmière de Prenato

8. Pour chacun des énoncés suivants, indiqué l’importance de chacune de ces raisons dans votre décision d’effectuer un test par ADN fœtal pour les trisomies communes.

	Pas important	Peu important	Neutre	Important	Très important
Je veux un enfant en bonne santé	○	○	○	○	○
Je veux les résultats le plus rapidement possible au cours de ma grossesse	○	○	○	○	○
Je veux la plus grande fiabilité des résultats en utilisant l’ADN fœtal	○	○	○	○	○
Je veux le plus d’information génétique possible à propos de la santé de mon enfant ou de ma santé	○	○	○	○	○
Je veux me préparer à la naissance d’un enfant avec une maladie génétique avant sa naissance si cela arrive	○	○	○	○	○
J’ai choisi ce test car il est facilement réalisé (une prise de sang seulement)	○	○	○	○	○
Le risque d’avoir recours à d’autres tests à la suite de celui-ci est mince, si les résultats sont satisfaisants	○	○	○	○	○
Ce test est sécuritaire et sans risque de fausse couche	○	○	○	○	○
L’ADN fœtal est le test qui a le plus de chance de trouver une condition présente	○	○	○	○	○

Information additionnelle qui pourrait être accessible grâce à l'ADN fœtal (trouvailles fortuites)

9. Par rapport aux trouvailles fortuites, quel genre d'information seriez-vous intéressée à recevoir ? (Vous pouvez cocher plusieurs possibilités)

- ☐ Je veux les informations qui pourraient avoir une répercussion immédiate sur la santé de mon bébé pendant ma grossesse
- ☐ Je veux les informations qui pourraient avoir une répercussion immédiate sur la santé de mon bébé dès sa naissance
- ☐ Je veux les informations qui pourraient avoir une répercussion future sur la santé de mon enfant même à l'âge adulte
- ☐ Je veux les informations qui pourraient avoir une répercussion immédiate ou future sur ma santé
- ☐ Je ne veux aucune information de ce genre, je ne veux que les informations concernant les trisomies communes 13, 18 et 21

10. Pour chacun des énoncés suivants, concernant la santé du bébé, indiquez l'importance que revêtirais celui-ci dans la prise de décision si vous feriez ou non le test de dépistage pour les trouvailles fortuite.

	Pas important	Peu important	Neutre	Important	Très important
Je veux savoir si mon enfant est atteint d'une maladie génétique	○	○	○	○	○
Je ne veux pas d'enfant atteint d'une maladie génétique	○	○	○	○	○
Je veux les résultats le plus rapidement possible au cours de ma grossesse	○	○	○	○	○
Je veux le plus d'informations possible à propos de la santé immédiate de mon enfant	○	○	○	○	○
Je veux le plus d'informations possible sur la santé futur de mon enfant	○	○	○	○	○
Je veux me préparer à la naissance d'un enfant avec une maladie génétique avant sa naissance	○	○	○	○	○
Je pense que je vais le regretter plus tard si je ne fais pas le test pour les trouvailles fortuites	○	○	○	○	○
Le coût additionnel pour le dépistage des trouvailles fortuites	○	○	○	○	○
L'anxiété générée par les résultats	○	○	○	○	○
Mes croyances religieuses	○	○	○	○	○
Ma sage-femme, mon médecin ou tout autre personnel de la santé pense que c'est une bonne idée de dépister pour ces trouvailles fortuites	○	○	○	○	○
Mon conjoint(e), ma famille ou mon environnement pense que c'est une bonne idée de dépister pour ces trouvailles fortuites	○	○	○	○	○

11. Puisque ces trouvailles fortuites pourraient comporter un risque personnel ou familial, indiquez l'importance que revêtirais chacun des énoncés suivant dans la prise de votre décision pour connaître ou non les informations relatives à votre santé

	Pas important	Peu important	Neutre	Important	Très important
Je veux le plus d'information possible à propos de ma santé	◯	◯	◯	◯	◯
L'anxiété généré par les résultats	◯	◯	◯	◯	◯
Mes croyances religieuses	◯	◯	◯	◯	◯
Le coût additionnel pour le dépistage des trouvailles fortuites	◯	◯	◯	◯	◯
Je pense que je vais le regretter plus tard si je ne fais pas le test des trouvailles fortuites	◯	◯	◯	◯	◯
Ma sagefemme, mon médecin ou un autre professionnel de la santé pense que c'est une bonne idée de dépister ces trouvailles fortuites	◯	◯	◯	◯	◯
Mon conjoint(e), ma famille ou mon environnement pense que c'est une bonne idée de dépister ces trouvailles fortuites	◯	◯	◯	◯	◯

12. Pour chacune des affirmations suivantes, indiquez dans quelle mesure vous vous sentiriez à l'aise avec ces découvertes fortuites pouvant impliquer un risque personnel ou familial.

	Pas confortable	Peu confortable	Neutre	Confortable	Très confortable
Obtenir des résultats qui donnent une évaluation des risques, plutôt qu'une réponse oui/non	◯	◯	◯	◯	◯
Dépister pour des conditions qui sont bien comprises	◯	◯	◯	◯	◯
Dépister pour des conditions qui ne sont pas bien comprises	◯	◯	◯	◯	◯
Dépister pour des conditions qui vont influencer les soins durant la grossesse	◯	◯	◯	◯	◯
Dépister pour des conditions qui ne vont pas influencer les soins durant la grossesse	◯	◯	◯	◯	◯
Dépister pour des conditions qui son traitables	◯	◯	◯	◯	◯
Dépister pour des conditions pour lesquelles il n'existe pas de traitement	◯	◯	◯	◯	◯
Si les troubles potentiels n'apparaissaient qu'à l'âge adulte	◯	◯	◯	◯	◯

13. Quel est votre sentiment par rapport à votre choix concernant le dépistage d'ADN fœtal

	Non	Un peu	Oui
Je me sens calme	◯	◯	◯
Je me sens stressée	◯	◯	◯
Je suis confuse	◯	◯	◯
Je suis bien	◯	◯	◯
Je suis inquiète	◯	◯	◯
Je suis satisfaite	◯	◯	◯

Prenato Patient Survey

Section sur les moyens financiers pour les dépistages par ADN fœtal et les trouvailles fortuites

14. Avez-vous des assurances qui couvrent les frais des tests génétiques par ADN fœtal ?

- Oui mes assurances couvrent la totalité des frais
- Oui mais seulement une partie des frais
- Non, je ne pense pas
- Non
- Je ne sais pas

Si oui, merci d’indiquer le nom de votre assurance ou organisme de protection :

15. Combien seriez-vous prêt à payer en plus de votre poche pour avoir accès aux tests de dépistages génétiques pour les informations supplémentaires (autres anomalies génétiques additionnelles ou trouvailles fortuites)?

- Moins de 100$
- 100 - 200$
- 201 - 300$
- 301 - 400$
- 401 - 500$
- Plus de 500$
- Le montant n’est pas important
- Je ne veux pas faire ce test

16. Pensez-vous que le régime public devrait couvrir les frais pour ce type de test de dépistage (ADN fœtal et les trouvailles fortuites)?

- Oui, ces tests devraient être remboursés pour tout le monde
- Oui mais seulement pour les personnes qui comporteraient un risque élevé
- Non je ne pense pas
- Je ne sais pas

17. Est-ce que le coût du test est un paramètre dans votre choix de dépistage?

- Non, de toute façon, je ne veux pas de test pour les informations génétiques additionnelles, seul le dépistage des trisomies communes m’intéresse
- Non, je peux me permettre le test que je veux
- Non, car mes assurances couvrent ces tests
- Oui, car je ne peux pas me permettre tous les tests disponibles
- Oui, car mes assurances ne couvrent pas ces dépistages additionnels
- Oui, Autre:

Prenato Patient Survey

Section socio-culturelle

18. Date de naissance (mois/année)

19. Quel est votre pays de naissance

20. Quelle est votre origine ethnique

- [ ] Caucasien
- [ ] Latino-Américain
- [ ] Asiatique
- [ ] Descendance africaine
- [ ] Moyen-oriental
- [ ] Première nation
- [ ] Autres :

21. Quelle sont vos origines religieuses/culturelles ?

- [ ] Bouddhiste
- [ ] Catholique
- [ ] Catholique orthodoxe
- [ ] Chrétien
- [ ] Juif
- [ ] Hindou
- [ ] Musulman
- [ ] Protestant
- [ ] Sikh
- [ ] Aucune affiliation religieuse
- [ ] Autre:

22. Quelle est l'importance de la religion/culture dans votre vie?

Pas important	Peu Important	Neutre	Important	Très important
○	○	○	○	○

23. Quelle est la langue le plus souvent utilisée à la maison ?

- Français
- Anglais
- Autre:

24. État civil

- Célibataire
- Mariée
- En couple mais non mariée
- Veuve
- Divorcée
- Autre:

25. Quel est le plus haut niveau d'étude pour lequel vous avez obtenu un diplôme?

- Diplôme d'étude secondaire
- Diplôme d'étude collégial
- Formation professionnelle
- Baccalauréat
- Maitrise
- Doctorat
- Autre:

26. Quel est votre occupation :

- À la maison
- Étudiante
- Travail à temps partiel
- Travail à temps complet

27. Quel est votre revenu annuel familial ?

- ◯ Aucun
- ◯ Moins de 10 000$
- ◯ 10 001$ à 20 000$
- ◯ 20 001$ à 50 000$
- ◯ 50 001$ à 100 000$
- ◯ 100 001$ à 300 000$
- ◯ Plus de 300 000$

28. d1. Êtes-vous un professionnel de la santé ?

- ◯ Non
- ◯ Oui

Si oui, précisez :
