# Supplementary material for: Patient attitudes and preferences about expanded noninvasive prenatal testing
Source: Front Genet. 2023 Apr 18;14:976051. doi: 10.3389/fgene.2023.976051 (PMC10161390; doi:10.3389/fgene.2023.976051)
Supplement: Supplementary file 3 [file DataSheet3.pdf]

Prenato Patient Survey

1. How many weeks pregnant are you?

2. Do you already have children?
   - ◯ Yes
   - ◯ No
   - ◯ If yes, how many:

3. During your previous pregnancies, did you undergo a fetal DNA screening?
   - ◯ Yes, for all my pregnancies
   - ◯ Yes, but not for all my pregnancies
   - ◯ No
   - ◯ Not applicable

4. Have you ever had a miscarriage or lost a baby?
   - ◯ No
   - ◯ Yes

5. This pregnancy was the result of:
   - ◯ Natural conception
   - ◯ In vitro fertilization (IVF)
   - ◯ Assisted reproductive technology other than (IVF)
   - ◯ Other:

6. Please indicate whether any of the following applies to you:

	No	Yes
The results of the first or second trimester ultrasound indicated that further genetic investigations were needed	○	○
I am going to be 35 or older at the time of delivery	○	○
I had a blood test that indicated that it was better to continue with genetic investigations	○	○
I had a previous pregnancy where there was a genetic abnormality	○	○
My partner or I have a chromosomal abnormality which increases our chances of having a child with chromosomal variations	○	○
My partner or I have a family history of chromosomal abnormalities	○	○
I have a condition that predisposes me to having a child with chromosomal abnormalities (if so, please specify)	○	○

Details:

Prenato Patient Survey

Fetal DNA screenings during pregnancy for the most common trisomies (Down's, Edwards' and Patau's syndrome)

7. What information would you like to receive through the fetal DNA test? (You can select more than one option)

- ☐ The sex of the child
- ☐ Screening for the most common trisomies (Down's, Edwards' and Patau's syndrome) only
- ☐ All trisomies including those involving rarer chromosomes (rare trisomies)
- ☐ All microdeletions.
- ☐ All genomic imbalances (also called CNV)
- ☐ Sex chromosome abnormalities because sometimes they affect the health of the baby.
- ☐ I want to obtain any genetic information that may affect the baby
- ☐ I am not sure yet and I will seek advice during my consultation with the Prenato nurse

8. For each of the following statements, indicate how important each reason was in your decision to have a fetal DNA test for common trisomies.

	Not important	Not very important	Neutral	Important	Very Important
I want a healthy child	○	○	○	○	○
I want the results as soon as possible during my pregnancy	○	○	○	○	○
I want the reliability of the results to be as high as possible by using fetal DNA	○	○	○	○	○
I want as much genetic information as possible about my child's health or my health	○	○	○	○	○
I want to be prepared before the birth if I am going to have a child with a genetic disorder	○	○	○	○	○
I chose this test because it is easily carried out (a blood test only)	○	○	○	○	○
If the results are satisfactory, there is very little possibility that further tests will be needed	○	○	○	○	○
This test is safe with no risk of miscarriage	○	○	○	○	○
Fetal DNA is the most effective test for finding these conditions	○	○	○	○	○

Prenato Patient Survey

Additional information that could be accessed through fetal DNA (incidental findings)

9. What sort of information would you be interested in receiving with regard to incidental findings? (You can select more than one option)

- ☐ I want to receive any information that could have an immediate impact on my baby's health during my pregnancy.
- ☐ I want any information that could have an immediate impact on my baby's health from birth
- ☐ I want information that could have a future impact on my child's health, even in adult life.
- ☐ I want information that could have a future or immediate impact on my health
- ☐ I don't want this sort of information, I only want information about common trisomies (Down's, Edwards', and Patau's syndrome)

10. For the following statements about the baby's health, please indicate how important each one would be in making a decision about whether or not you would be screened for incidental findings.

	Not important	Not very important	Neutral	Important	Very Important
I want to know if my child has a genetic disorder					
I do not want a child that has a genetic disorder					
I want the results as soon as possible during my pregnancy					
I want as much information as possible about my child's immediate health					
I want as much information as possible about my child's future health					
I want to be prepared before the birth if I am going to have a child with a genetic disorder					
I think I will regret it later if I don't undergo the test for incidental findings					
The additional cost of screening for incidental findings					
Anxiety generated by results					
My religious beliefs					
My midwife, doctor, or other health care staff think it's a good idea to screen for these incidental findings					
My spouse, family, or environment think it's a good idea to screen for these incidental findings					

11. Since these incidental findings may pose a personal or family risk, please indicate how important each of the following statements would be in making the decision about whether to obtain information regarding your health

	Not important	Not very important	Neutral	Important	Very Important
I want as much information as possible regarding my health	○	○	○	○	○
Anxiety generated by the results	○	○	○	○	○
My religious beliefs	○	○	○	○	○
The additional cost of screening for incidental findings	○	○	○	○	○
I think I will regret it later if I don't undergo the test for incidental findings	○	○	○	○	○
My midwife, my doctor, or another health professional thinks it's a good idea to screen for these incidental findings	○	○	○	○	○
My spouse, family, or environment thinks it's a good idea to screen for these incidental findings	○	○	○	○	○

12. For each of the following statements, please indicate how comfortable you would be with incidental findings that may involve personal or family risk.

	Not comfortable	Not very comfortable	Neutral	Comfortable	Very comfortable
Get results that give an assessment of the risks, rather than just a “yes/no” answer	○	○	○	○	○
Screen for conditions that are well known	○	○	○	○	○
Screen for conditions that are not well known	○	○	○	○	○
Screen for conditions that will influence care during pregnancy	○	○	○	○	○
Screen for conditions that will not influence care during pregnancy	○	○	○	○	○
Screen for conditions that are treatable	○	○	○	○	○
Screen for conditions for which there is no treatment	○	○	○	○	○
If potential disorders do not appear until adulthood	○	○	○	○	○

13. How do you feel about your choice regarding fetal DNA testing?

	No	A little	Yes
I feel calm	○	○	○
I feel stressed	○	○	○
I'm confused	○	○	○
I'm fine	○	○	○
I'm worried	○	○	○
I'm satisfied	○	○	○

Prenato Patient Survey

Section on financial resources for fetal DNA screenings and incidental findings

14. Do you have insurance that covers the cost of fetal DNA genetic testing?

- ○ Yes, my insurance covers all the costs
- ○ Yes, but only part of the costs
- ○ No, I don't think so
- ○ No
- ○ I don't know

If so, please include the name of your insurance or protection agency:

15. How much would you be willing to pay to access genetic screening for additional information (other genetic abnormalities or incidental findings)?

- ○ Less than $100
- ○ $100–200
- ○ $201–300
- ○ $301–400
- ○ $401–500
- ○ More than $500
- ○ The amount is not important,
- ○ I do not want to have this test

16. Do you think the public health plan should cover the costs for this type of screening test (fetal DNA and incidental findings)?

- ◯ Yes, these tests should be free for everyone
- ◯ Yes, but only for people who are high risk
- ◯ No I don't think so
- ◯ I don't know

17. Is the cost of testing a factor in your screening decision?

- ◯ No, I don't want a test for additional genetic information, I'm only interested in screening for trisomies (Down's, Edwards', and Patau's syndrome)
- ◯ No, I can afford the test I want
- ◯ No, because my insurance covers these tests
- ◯ Yes, because I can't afford all the tests available
- ◯ Yes, because my insurance does not cover these additional screenings
- ◯ Yes, Other:

Prenato Patient Survey

Socio-cultural section

18. Date of birth (month/year)

19. What is your country of birth?

20. What is your ethnicity?

- [ ] Caucasian
- [ ] Latin American
- [ ] Asian
- [ ] African descent
- [ ] Middle Eastern
- [ ] First Nation
- [ ] Other:

21. What is your religious/cultural background?

- [ ] Buddhist
- [ ] Catholic
- [ ] Orthodox Catholic
- [ ] Christian
- [ ] Jewish
- [ ] Hindu
- [ ] Muslim
- [ ] Protestant
- [ ] Sikh
- [ ] No religious affiliation
- [ ] Other:

22. How important is religion/culture in your life?

Not important	Not very important	Neutral	Important	Very Important
○	○	○	○	○

23. What is the language most often used at home?

- ○ French
- ○ English
- ○ Other:

24. Civil Status

- ○ Single
- ○ Married
- ○ In a relationship but not married
- ○ Widow
- ○ Divorced
- ○ Other:

25. What is your highest level of education?

- ○ High school diploma
- ○ College degree
- ○ Professional training
- ○ Baccalaureate
- ○ Master's degree
- ○ Doctorate
- ○ Other:

26. What is your occupation:

- ○ Homemaker
- ○ Student
- ○ Part-time employment
- ○ Full-time employment

27. What is your annual family income?

- None
- Less than $10,000
- $10,001 to $20,000
- $20,001 to $50,000
- $50,001 to $100,000
- $100,001 to $300,000
- More than $300,000

28. Are you a health care professional?

- No
- Yes

If so, please specify:
