# Supplementary material for: Patient attitudes and preferences about expanded noninvasive prenatal testing
Source: Front Genet. 2023 Apr 18;14:976051. doi: 10.3389/fgene.2023.976051 (PMC10161390; doi:10.3389/fgene.2023.976051)
Supplement: Supplementary file 4 [file DataSheet1.pdf]

Welcome to Prenato and thank you for agreeing to complete this short questionnaire which we need to get to know our patients better in order to offer a service that is always at the forefront of modern medicine and, above all, listening to you.

Genetic screening test and diagnostic test

A genetic prenatal screening test aims to identify babies at significant risk of carrying a genetic disease. The results obtained during a screening test do not give a definitive answer, but they make it possible to identify patients at risk for whom additional diagnostic testsare indicated to determine with certainty whether the child is affected by a chromosome abnormality.

Diagnostic tests that confirm, beyond a reasonable doubt, the presence or absence of genetic abnormalities are usually done by analyzing the baby’s cells after amniocentesis, for example. It is the result of these diagnostic tests that will determine with certainty the genetic health of the baby. However, these diagnostic tests are associated with a certain risk of complications for the baby and that is why this procedure is reserved for women identified to be at risk during screening tests.

Genetic diseases

Most children born in Quebec are in good health. However, a small proportion are born with genetic health problems that can sometimes be detected during pregnancy by different tests.

Usually, each human cell has 23 pairs of chromosomes for a total of 46, of which the last pair is made up of sex chromosomes (XX for a woman and XY for a man). Genetic diseases can be the result of an abnormality in the number of these chromosomes (one more or less chromosome making a trisomy or a monosomy) or sometimes an abnormality in the structure of one of these chromosomes, by the loss of a chromosome segment (deletion) or even the addition of only part of a chromosome (duplication producing a partial trisomy).

The most common and well-known anomalies are trisomies 13, 18 and 21 as well as anomalies in the number of sex chromosomes:

Sometimes a chromosome can be screened that is not part of the common trisomies. These so-called rare trisomies can also be important because they can have a poor prognosis if they are present in the baby, or identify a risk for the pregnancy (ex: growth issues) if they are present in the baby's placenta. The prognosis associated with these trisomies is less well known than that of common trisomies.

Finally, certain genetic syndromes are also available for prenatal screening. These correspond to an imbalance for a segment of a chromosome producing a genomic imbalance with a consequent risk of a child with a genetic deficiency.

Fetal DNA testing

Fetal DNA testing uses DNA from the fetus in the mother's blood to quantify the number of chromosomes. At the time of fetal DNA screening, parents or health professionals requesting the test are completely free to choose the type of screening that will be carried out on the mother's blood sample. Thus, the fetal DNA test could:

- Only screen for common trisomies (chromosomes 13, 18 and 21).
- Include the identification of the sex of the baby and screening for abnormalities of the sex chromosomes.
- Also look for the rarer trisomies, concerning the other chromosomes that can have consequences on the progress of the pregnancy or the health of the baby.
- Analyze the structure of certain chromosomes for microdeletion syndromes associated with a risk of health and developmental issues in the child at birth.

Incidental findings

Generally, all the genetic abnormalities offered for screening and mentioned above will result in visible disorders of the baby during pregnancy or at birth. However, in some cases, the symptoms of a genetic abnormality detected during pregnancy could only develop during childhood, or even in late adulthood, with the possibility of medical treatment or not. These situations, with the rapid advance of modern genetics, should become more frequent in the future, which explains some of the questions in our questionnaire because your opinion is essential, and it is very important to listen to parents in order to best adapt the tools of tomorrow's medicine.
